# Romantic Competence and Adolescent Courtship: The Multidimensional Nature of the Construct and Differences by Age and Gender

**DOI:** 10.3390/ijerph17145223

**Published:** 2020-07-20

**Authors:** Carmen Viejo, Noemí Toledano, Rosario Ortega-Ruiz

**Affiliations:** Department of Psychology, University of Córdoba, 14071 Córdoba, Spain; cviejo@uco.es (C.V.); ed1orrur@uco.es (R.O.-R.)

**Keywords:** adolescence, romantic competence, erotic-affective interest, questionnaire validation

## Abstract

Adolescent courtship is emerging as an important developmental process which impacts social balance and adjustment in the teenage years. Both the cultural context and different individual competencies seem to determine the success or failure of this process. However, there is little research focusing on the direct relationship between interpersonal skills and adolescent courtship, possibly due to the lack of suitable instruments to measure it. This study takes this process further by adapting a multifactorial measurement of Interpersonal Competence to the framework of adolescent courtship (Adolescent Interpersonal Competence Questionnaire for Courtship (AICQc)), and by analyzing these skills according to gender and age. A total of 1584 adolescents (48.9% girls and 51.1% boys) between the ages of 12 and 17 who were in compulsory secondary education participated in the study. Based on the factor model proposed by Buhrmester et al., the Confirmatory Factor Analysis showed the validity of the instrument and a high internal consistency for five independent domains of competence: (a) initiating relationships; (b) assertiveness and the ability to say no; (c) self-disclosure; (d) providing emotional support; and (e) resolving conflicts. Age, as measured by the school year, was found to be a key factor in this regard. The results are discussed in terms of assessing interpersonal competence for relationships. There has been little research into this type of interpersonal competence and it is a key factor in facing the important developmental task for first-time couples of choosing a partner and managing adolescent courtship.

## 1. Introduction

### 1.1. Relationships and Adolescent Courtship

All youngsters become interested in initiating and forming romantic relationships during adolescence as an intrinsic part of their personal development [1,2]. For most boys and girls, this process has its origin in the network of social relationships they enjoy with their peers [3], and this obviously involves initiating interpersonal relationships which contain a certain degree of intimacy and affectivity. It should be seen, therefore, as a specific developmental task, since romantic relationships have a direct influence on some basic psychosocial aspects of adolescent development such as the formation of self-concept during adolescence [4] or the development of intimacy and trust with in the romantic relationships [5]. This developmental task begins in the early years of adolescence and can carry on into young adulthood, with varying degrees of success on a personal level or as couples.

Indeed, the evolution of peer networks, added to the onset of puberty and psychological development which occurs at these ages, means that boys and girls at this stage establish a wide spectrum of interpersonal relationships differentiated in terms of their defining characteristics and needs, ranging from acquaintances to friends, close friends and the first romantic relationships [6]; when erotic and affective variables are added to this continuum, the relationships become more complex and result in the beginning of the courtship process, which includes flirting and dating. If successful, they could, in turn, lead progressively to the desire to form relationships with a higher degree of intimacy and emotional support, which result in the first romantic relationships [7,8].

From a psycho-developmental perspective, this process constitutes a psycho-developmental challenge for the teenagers [9], in which different processes of social competence are at play [10]. The first erotic-sentimental relationships should be considered as important learning experiences for boys and girls, providing a new framework for the security and support which adolescents crave [11], and contributing to their overall well-being [12,13]. The emergence of feelings of intimacy, support and care which accompany the new courtship relationship, seems to trigger previously acquired interpersonal relationship strategies, such as recognizing emotions in oneself and others, negotiation and moral reciprocity. However, analyzing the origins of romantic relationships and the courtship process leading up to a choice of partner is a complex task [14]. The task of choosing and feeling chosen is not a straightforward one, as it involves previously acquired emotional, cognitive and social competences which are now upgraded to perform a different task. These characteristics mean that the courtship’s process and the formation of the first romantic relationships have an important impact on the psychosocial adjustment of adolescent boys and girls [15]. Although some studies with adolescents identify some problems, such as depression [16,17], overall, initiating a romantic relationships can be positive for the adjustment of adolescents and the development of romantic competence since it allows them to put unto practice important skills for the development of this competence such as negotiation, conflict management of giving emotional support to the partner [18,19].

Boys and girls display a diverse range of behaviour in initiating their approach to another person, demonstrating their interest in them or revealing their affections. They are not always effective, and these attempts, successful or not, may occasionally include risk factors for the courtship process [20]. In due course, the awakening of erotic interest lends the added function of attracting the attention of the chosen person to initiate a sentimental bond [21]. In the social context of peers, relational dynamics sets in motion as series of role plays and leadership disputes for popularity within the group, which in turn play a key role in the choice. In addition, the use of low-intensity aggressive behaviour [22] can be observed, especially in males, which can be interpreted either as clumsiness or as a desire for dominance, although this process has not been studied in depth [20]. In other words, the incipient nature of these relationships and the inexperience of their protagonists could mean that the process of courtship sometimes proceeds in a rash, awkward fashion, with relational patterns appearing which are not ideally suited to the new context of the first erotic-sentimental relations.

Individual and interpersonal skills previously used in the context of friendships provide, in preadolescence and adolescence, the necessary social competencies for initiating romantic relationships [7,23,24].In addition, the relationships that parents have with their children influences the development of adolescent romantic relationships and the development of romantic competence, in the sense that a warm and affective relationships between parents and children is positively related to the development of romantic competence and quality adolescent relationships [18].However, the specific, complex and multifaceted nature of how these erotic-sentimental relationships are initiated and maintained makes it difficult to pinpoint precisely which variables influence the appropriate handling of the adolescent courtship process [5,25].

### 1.2. Measuring Interpersonal Competence in the Adolescent Courtship Process

The complex task of defining a temporal limit for the courtship process has contributed to the fact that few works have specifically addressed how it can be measured. A number of benchmarks do exist, ranging from the Measure of Adolescent Heterosocial Competence [26], designed to assess the competence in establishing intimate relationships only with close friends, to the Relationship Self-Concept Questionnaire [27], designed to evaluate already established couples through variables such as intimacy, sexual behaviour or authenticity in their romantic encounters, but omitting aspects related to how the relationship is initiated [28].

One of the instruments developed most recently to evaluate romantic competence in the courtship process of adolescent boys and girls is the Romantic Competence Interview (RCI) [25]. This qualitative instrument was designed to validate the romantic competence model proposed by its authors, which is based on three skill domains: (a) insight, an awareness which recognizes in oneself and in the other person an interest or desire for the relationship; (b) mutuality, the ability to take the needs of the other person into account, respecting and valuing their opinions and factoring them into mutual decision making; and (c) emotional regulation, the ability to modulate and express emotions in response to experiences. According to the authors, the specific variables considered within each blocks can vary according to the couple stage; even though the insight, the mutuality and the emotional regulation are important from the beginning of the romantic relationship, during the flirting and dating process, to the married or well-establishedrelationship [19]. Although this instrument has great potential in terms of focusing on the courtship process and initiating relationships, the format of the unstructured interview makes it difficult to interpret and generalize the results. The Adolescent Interpersonal Competence Questionnaire (AICQ) [5,29] overcame these difficulties and, although not defined as a means of evaluating the courtship process, it has now been recognized as the one of the most widely used questionnaires to evaluate interpersonal adolescent relationships in couples of the same or different sexes, taking into account the initiation process in these relationships. According to some studies, although the AICQ makes only a passing reference to partners, the first heterosexual approaches it refers to have been identified with the first erotic-sentimental approaches that take place in adolescence [5]. The instrument is based on the Interpersonal Competence Questionnaire(ICQ) [30], which was aimed at adults and was designed to assess the level of self-perceived competence in same-sex friendships and romantic relationships with the opposite sex in five domains: (1) initiating relationships; (2) self-disclosure or the ability to disclose personal information to others; (3) the ability to be assertive and say ‘no’ to others; (4) the ability to provide emotional support and advice when another person is experiencing problems; and (5) the ability to resolve interpersonal conflict. In the later version for adolescents, the authors propose assessing the level of competence in establishing and maintaining intimate friendly relationships with peers of the same and different sex. To achieve this, some items were reformulated, particularly in the domain of conflict resolution, one of the original domains was excluded (assertiveness and the ability to say ‘no’) and a new domain added: the ability to influence others. These modifications, although they help to adapt the questionnaire to the target population, omit a few essential aspects, such as the crucial negotiation processes which occur during the courtship process as part of the approach to the other person.

### 1.3. The Present Study

This study has two main objectives: first, to present a quantitative instrument of evaluation which will allow us to measure the level of interpersonal competence in the context of the adolescent courtship process, viewed from a broad, multifactorial perspective. To achieve this, we used the versions of the Interpersonal Competence Questionnaire for adults (ICQ) [30] and for adolescents (AICQ) [5]; we also adapted the questionnaire to include the dimensions present during the process of approaching and initiating romantic relationships in adolescents, translating it into Spanish and validating it. The secondary aim of the study is to use this instrument to analyze the level of interpersonal competence in adolescent boys and girls which they draw on during the process of courtship and establishing their first romantic relationships, identifying any possible differences bygender or age.

## 2. Methods

### 2.1. Participants

The participants were 1584 adolescents (48.9% girls, 51.1% boys) who were pupils at 30 state schools in the Autonomous Community of Andalusia (Spain), of middle socioeconomic level, andwhose ages ranged between 12 and 17 (M = 13.56; DT = 1.21). 35% of the participants were in the 1st year of ESO (Secondary School), 30.7% were in their 2nd year, 30.4% in 3rd and 3.9% in 4th.

### 2.2. Instruments

We designed an evaluation tool composed of:

- Sociometric data: questions referring to participants’ personal variables (gender, age, school and course).
- The *Adolescent Interpersonal Competence Questionnaire for Courtship* (hereafter, AICQc): This evaluation tool was created to measure the level of competence shown by adolescents in their courtship relationships. This scale is an adaptation of the Interpersonal Competence Questionnaire in the version for adults (ICQ) [30] and for adolescents (AICQ) [5].After its adaptations and validation, the AICQc consisted of 35 items which describe everyday interpersonal situations and fall under five different domains: initiating relationships (α = 0.81), assertiveness and the ability to say ‘no’ (α = 0.84), self-disclosure (α = 0.81), giving emotional support (α = 0.88) and resolving interpersonal conflict (α = 0.86). The AICQc is measured on a 5-point Likert scale ranging from (1) “I feel awkward with this” to (2) “I can’t do this very well”, (3) “I can do this OK”,(4) “I’m fairly good at this” and (5) “I’m very good at this”. At the beginning of the questionnaire information was provided on how to use the 5-point scale, knowing that 1 = I feel awkward, meaning “I would feel uncomfortable and unable to handle the situation and would avoid it if possible”; and 5 = I am very good at this, meaning “I would feel very comfortable and could handle the situation very well”. The global index of romantic competence can be calculated with the standardized scores. The average level of total score of self-perceived competence was high (α = 0.96).

### 2.3. Design and Procedure

We adopted a cross-sectional, ex-post facto design for the study, with a single group. The schools and classes which took part were selected using an incidental design [31]. Ethical approval for the study was obtained from the Comité de Bioética y Bioseguridad de la Universidad de Córdoba (Bioethics and Biosafety Committee of the University of Cordoba) and developed in accordance with the considerations of the Declaration of Helsinki and the Spanish Society of Psychology. The study was approved by the school boards, and the consent obtained from the parents of the participants was both written and informed. The questionnaire took approximately 30 min to complete.

### 2.4. Data Analysis

To test the structure of the AICQc in the five domains, a confirmatory factor analysis (CFA) was carried out, based on the theoretical models of Furman and Buhrmester [5,29,30], but only using complete cases (N = 1032). A polychoric correlation matrix was used to carry out the initial reliability analysis to check the mean inter-item correlation. Robust maximum likelihood (ML) estimation methods were used and the variables were specified as categorical, since the AICQc was measured on a Likert-type response scale. The value of 366,000 obtained for the Mardia coefficient showed that the population did not meet the multivariate normal distribution criteria [32].

To assess the suitability of the model, the Satorra-Bentler Chi-square, Bentler-Bonet non-standard fit index (NNFI) (≥0.90 adequate, ≥0.95 optimal) and the mean square error (RMSEA) (≤0.08 adequate, ≤0.05 optimal) were taken into account, adjusted to the values of the comparative adjustment index (CFI) [33]. In addition, following [34,35], the convergent validity was assessed by reporting the standardized factor weights, and the internal validity was estimated by calculating the alpha value for each factor and the ordinal alpha of the questionnaire.

The statistical software EQS 6.2 (Multivariate Software, Inc., Encino, CA, USA) was used to carry out all the analyses. For the second main objective of the study, descriptive analyses were carried out with the total number of participants (*N* = 1584) using SPSS 20.0 statistical software (IBM, Armonk, NY, USA). Analyses of comparison of means in relation to the participants’ gender and school year were performed using Student’s t-test and ANOVA, respectively. Due to the sample sizes, the Games-Howell procedure was used for the post hoc test [36]. The size of the effect was controlled using Cohen’s d and Eta-square tests (low effect r = 10; medium effect r = 0.30; high effect r = 0.50) [37].

## 3. Results

### 3.1. Adaptation and Validation of the AICQc—The Interpersonal Competence Questionnaire in the Context of Adolescent Courtship

To configure the AICQc, the two original instruments, ICQ and AICQ, were subjected to a back-translation process, which enabled us to use the Spanish versions.

The scales which referred to initiating relationships, self-disclosure, giving emotional support and resolving conflicts were used from the AICQ, for two reasons: (1) the version for adolescents refers to partners of the same and different sexes, thus alluding to the first erotic-romantic approaches [7]; (2) the items are suitably adapted to the age of the adolescents and the domains can be adapted to the courtship process. In our version, the aspects omitted from the adolescent version were: the scale of “influencing the other person”, because its items presupposed a pre-existing romantic relationship, which did not fit in with the aim of the study. However, the “assertiveness and ability to say ‘no’” scale from the adult version was added, and the way the items were expressed was adapted whenever the wording required it. Items which referred to “*the person you like*” or “*the person you start dating*” were reformulated, referring to the process of courtship, regardless of sexual orientation.

This first formulation of the questionnaire resulted in a 36-item instrument which was then subjected to a validation process. The first estimated AFC showed positive results, with good adjustment indices (S-Bχ^2^ = 2377.5639; *p* = 0.00; NNFI = 0.982; CFI = 0.983; RMSEA = 0.055 (90% CI [0.052, 0.057]). However, the values for the Chi-square test, factor weight and measurement error for the item “*Do you feel you are able to control your temper when arguing with someone you like or who you have started to go out with?”* (r^2^ = 0.230; 0.479f5 + 0.878 e33) showed that this item had a poor fit in the model, so a new model was estimated omitting this item, which produced a 35-item scale which was then tested in a five-domain model (see Table A1). The structure of the model reflected the version proposed by Furman and Buhrmester [5,29,30]. The internal correlation analyses showed a minimal relationship between items and low collinearity (Table 1).

**Figure 1:**
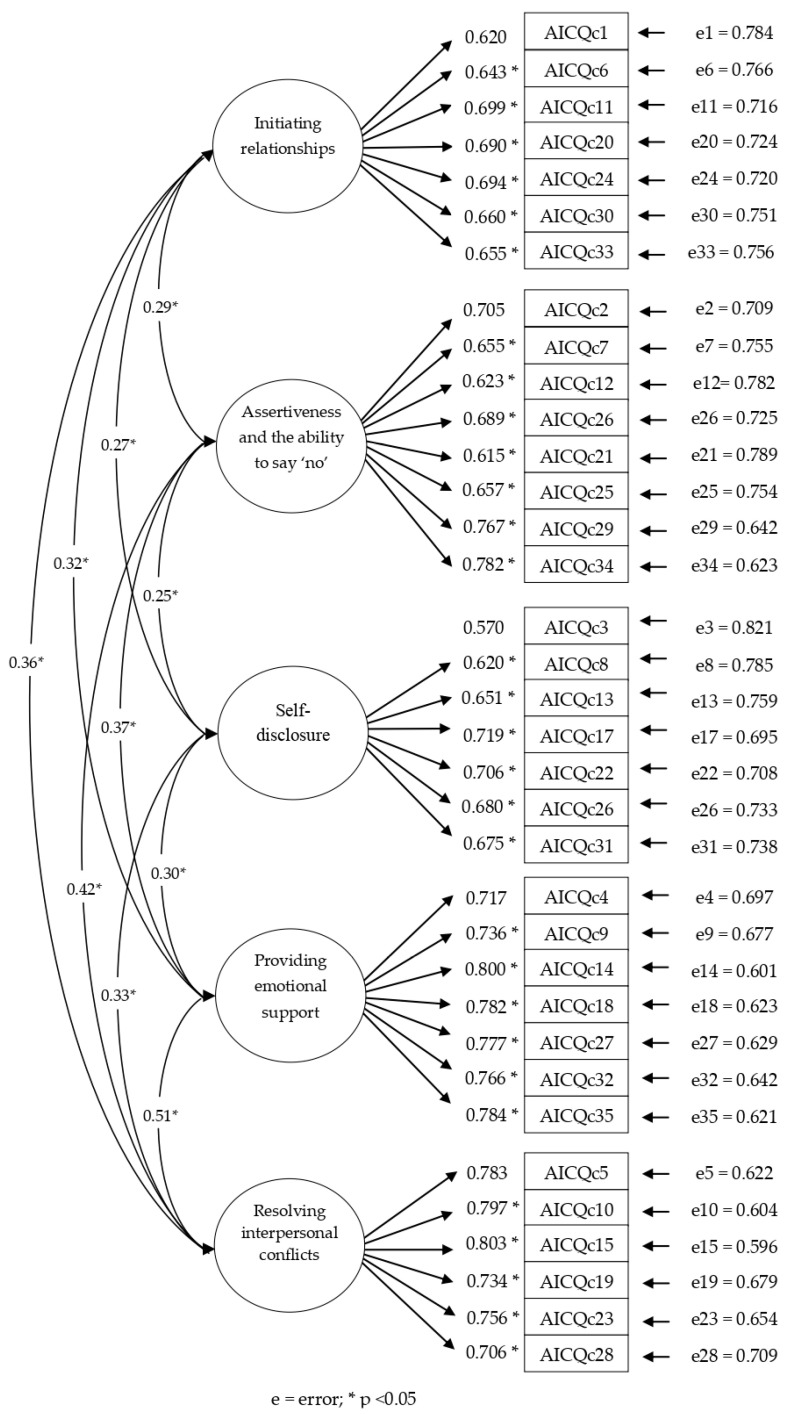
Standardized solutions for the Confirmatory Factor Analysis.

The AFC now showed that the model fitted correctly (S-Bχ^2^ = 2287.0483; *p* = 0.00; NNFI = 0.983; CFI = 0.984; RMSEA = 0.055 (90% CI [0.053, 0.058]). The model parameters are shown in Figure 1.

The overall reliability of the scale was 0.96. The alpha value for the five subscales showed high values (initiating relationships α = 0.81; assertiveness and ability to say ‘no’ α = 0.84; self-disclosure α = 0.81; giving emotional support α = 0.88; resolving interpersonal conflict α = 0.86). The scale showed good convergent validity, with values for standardized factorial weights greater than 0.57. The values for covariances were low and significant, confirming the structure of the model in five independent domains.

### 3.2. Interpersonal Competence in the Courtship Process

The secondary aim of this work was to analyze the level of interpersonal competence that adolescents show in their courtship relationships, verifying whether there are any differences by gender and school year in the five competency domains.

The results showed average scores for the five AICQc domains: initiating relationships (M = 14.29; DT = 5.51), assertiveness and the ability to say ‘no’ (M = 17.75; DT = 6.39), self-disclosure (M = 12.53; DT = 5.62), giving emotional support (M = 17.38; DT = 5.60) and conflict resolution (M = 16.10; DT = 5.65), with *assertiveness and the ability to say ‘no’*, and *the ability to give emotional support* the domains which obtained the highest scores (Table 2).

The analyses which compared the averages by gender showed significant differences only for the factor of giving emotional support (t (1251) = −4628; *p* = 0.026; d = 0.26), for which it was seen that girls (M = 18.12; DT = 5.35) gave more emotional support than boys (M = 16.68; DT = 5.72) in the process of approaching and courting the other person, although the effect size was small (Table 3).

As regards age, which was measured by school year, there were also significant differences in the scales of assertiveness and the ability to say ‘no’ (*F* (4, 1249) = 2.63; *p* = 0.03; *η*^2^ = 0.008), self-disclosure (*F* (4, 1252) = 2.55; *η*^2^ = 0.008) and the ability to give emotional support (*F* (4, 1276) = 2.79; *p* = 0.02; *η*^2^= 0.009). Post-hoc tests showed that, in all cases, the adolescents in the higher years (4th year of Secondary—between 16 and 17 years old) obtained significantly higher average scores than boys and girls in lower years (1st/2ndyear).

## 4. Discussion

Despite being identified as a key psycho-developmental task, there has been little research into romantic advances and the initiation of courtship as an erotic game of initiation in forming a couple or as an incipient phase of the search for intimacy among adolescents. However, adolescents and young people enjoy a rich, intense emotional life and this seems to occupy a key role in their well-being [12]. From a psycho-developmental perspective, this process involves a continuum which ranges from friendly relationships with peers, through the attraction to and intimacy with some friends more than others, to the establishment of the first relationships as a couple [6,38]; in other words, the process of courtship lays the foundations for establishing future relationships as a couple, which opens up a new framework for development and learning which will continue throughout the subsequent years [39].For this reason, it is vital to identify which variables modulate this process, and to what extent they condition the success or failure of these first sentimental experiences, and perhaps, wherever possible, clarify the nature of a social learning experience which has a major impact on the future of adolescent boys and girls [7]. However, the lack of instruments to analyze this process has hindered scientific access to this field. This work focuses on this lack of measurement instruments and its primary aim was to devise, by adapting, validating and adjusting earlier instruments, a robust, validated psychometric questionnaire which would shed greater light on the subject.

The results obtained for the adaptation of the AICQc showed not only that the adjusted questionnaire was suitable, but also that it had great potential in two key areas: (a) to focus specifically on the exact competencies established during teenage courtship: although these relationships may be at first be unstable, within them lie processes of seeking intimacy and mutual support which, together with the erotic impulse, make these courtship processes thrive, despite the fact that they are not yet well understood by science. This amalgam of choices, feelings, desires, attitudes and behaviour, despite its complexity, is fully recognizable by the protagonists; and (b) to identify the key factors of competencies, motivation, empathy and social adjustment which are activated in these processes [10], because they seem to form the basic competency for initiating new romantic experiences [10,20,23,24] at an age when the search and choice of an erotic-romantic partner is another key element in the development process. The step that involves moving on from a relationship of friendship to an erotic-sentimental relationship requires boys and girls to be able to develop, transform and articulate a number of skills which they previously either did not possess, were used for other purposes or functioned independently. The complexity of the process of courtship means that a specific, multifaceted outlook is required in order to understand the meaning of these approaches and the skills they require to carry it out successfully [5,27,30,40,41].

The instrument adapted and validated in this work essentially meets the requirements, which constitutes the second great potential of this questionnaire: the interpretation of interpersonal competition in this context as a multidimensional construct containing five independent but interconnected domains of social competence: the ability to initiate relationships, assertiveness and ability to say ‘no’, the ability to self-disclose, the ability to give emotional support, and the ability to resolve interpersonal conflict.

From a psycho-developmental perspective, it must be considered that, just like any learning and development process, the ability to perform the tasks involved in the courtship process and the beginning of the first romantic relationships can vary considerably depending on gender and age [42,43]. For this reason, the second main aim of this study was to analyze the level of interpersonal competence existing in the framework of adolescent courtship among adolescents, while taking into account the possible differences of gender and school year.

As regards gender, previous results have been controversial: some studies have shown that the differences are minimal and, in general terms, that boys and girls are equally competent or incompetent in their romantic relationships [7]; others report that boys and girls prove to be equally competent in their relationships of friendship [43], but that girls seem to have greater capacity in certain aspects such as intimacy or the ability to communicate with the other person [25], as well as in skills that require specific expressive competencies such as self-disclosure or giving emotional support [5,30]. The results of our study have revealed that although, in general, boys and girls may present similar competency levels, there are differences in particular domains. Both girls and boys have a similar level of interpersonal competence in tasks such as starting relationships, assertiveness and the ability to say ‘no’, self-disclosure and resolving interpersonal conflict during the adolescent courtship process; however, girls are more skilled in providing emotional support to the chosen partner during courtship.

Regarding age, the results of this work indicate that, as shown in other investigations [14], interpersonal competence in adolescent courtship varies between the first phase (12–14 years old) and second phase (15–17 years old) of adolescence. An interesting topic for discussion is how to interpret and attribute meaning to this developmental marker. In this regard, some studies consider that from the age of 12 to 18, it is the general evolution of cognitive skills for understanding the social world that determines general maturity levels and, therefore, enhances the ability to manage communication, emotional control, assertiveness and relationships with other people. However, in turn, successful experiences in these initial courtships, and the lessons which are learnt from them, can also be interpreted as factors which impact and stimulate change. There is no doubt that the level of self-perceived competence around the age of 16 can make an important difference, as stated by Collins (2003) [14]. However, it is equally certain that the affective-communicative experience of these incipient courtship relationships provide a stimulus for the development of more advanced capacities such as emotional regulation, empathy or assertive communication, among others [44], which would also result in increased competencies in erotic-sentimental relationships.

On the other hand, studies in the tendencies towards risk in adolescents also support the idea that at the age of 15–16 years old, there seems to be a de-escalation in the trend towards the risk behaviour which is more typical in adolescents in the first stage of adolescence [45,46]. This could at least begin to explain how the emotional and relational challenges involved in courtship are addressed with greater competence in the second stage of adolescence, and that relationships are approached with more caution and self-perception of the emotional involvement and commitment to the other person which is required. They also recognize that the relationship includes elements of intimacy, such as the awareness of one’s own and another person’s emotions, the consideration of another person’s needs, giving help, the competence to perceive and resolve conflicts of interest, revealing emotions, and so on [25,44]. In any case, cognitive maturity, in this regard, has a key role to play in the point of inflection between these two sub-stages [47], which accounts for the fact that the three competency domains which produce higher scores in the second phase of adolescence are those of *self-disclosure, assertiveness and the ability to say ‘no’,* and *giving emotional support.*

### Limitations of the Study

This study has a number of limitations, and further research is required to advance in the study of adolescent courtship. Firstly, despite the fact that this work uses an adaptation of a previously validated instrument and that the domains have been operationally defined, the AICQ has been adapted to the context of the adolescent courtship process, and therefore an analysis of the content of the questionnaire items would give the contract greater relevance and generalizability [48]. Secondly, given the developmental nature of these abilities within the continuum of the courtship process, further longitudinal studies would allow us to evaluate the way these competences change and evolve, and to contrast them with other aspects of psychosocial functioning, such as the quality of the relationship or the satisfaction of both members during the courtship process, self-esteem, school achievement and establishment of the first romantic relationships; and with criteria of maladjustment such as depression or anxiety.

## 5. Conclusions

This work highlights the importance of scientifically supporting the idea that the courtship process is a specific psycho-evolutionary task that generally occurs in the course of adolescence, and therefore requires special attention. The specific and multifaceted character of the courtship process helps us to establish a unique model of romantic competence, also specific and multifaceted. In this sense, the five skills of romantic competence that have been highlighted in this paper would be used in order to improve the relational functioning of adolescents based on healthy functioning. In addition it is clear that the results presented here are relevant at the level educational intervention level. Adolescence seems to be a key period in which the interpersonal skills which help boys and girls to grow up as competent individuals in interpersonal relationships are developed and improved. These studies pave the way for designing educational procedures which can help to make the sentimental life of adolescents more positive, well-balanced and satisfactory [23,24].

## Figures and Tables

**Table 1:** Polychoric correlation matrix for the AICQc.

	i1	i2	i3	i4	i5	i6	i7	i8	i9	i10	i11	i12	i13	i14	i15	i16	i17	i18	i19	i20	i21	i22	i23	i24	i25	i26	i27	i28	i29	i30	i31	i32	i33	i34	i35
**i1**	1																																		
**i2**	0.45	1																																	
**i3**	0.37	0.27	1																																
**i4**	0.36	0.43	0.29	1																															
**i5**	0.45	0.52	0.38	0.64	1																														
**i6**	0.30	0.33	0.23	0.39	0.46	1																													
**i7**	0.20	0.43	0.07	0.32	0.30	0.30	1																												
**i8**	0.19	0.27	0.37	0.37	0.39	0.33	0.22	1																											
**i9**	0.33	0.44	0.27	0.55	0.59	0.41	0.38	0.51	1																										
**i10**	0.38	0.48	0.27	0.57	0.67	0.46	0.40	0.44	0.66	1																									
**i11**	0.39	0.34	0.22	0.40	0.47	0.61	0.34	0.32	0.43	0.50	1																								
**i12**	0.20	0.42	0.12	0.28	0.30	0.29	0.56	0.23	0.34	0.39	0.32	1																							
**i13**	0.33	0.27	0.49	0.23	0.37	0.20	0.17	0.47	0.32	0.35	0.29	0.23	1																						
**i14**	0.32	0.43	0.27	0.60	0.62	0.41	0.36	0.44	0.63	0.67	0.49	0.35	0.35	1																					
**i15**	0.33	0.44	0.24	0.57	0.63	0.44	0.42	0.40	0.61	0.66	0.47	0.39	0.34	0.71	1																				
**i16**	0.17	0.46	0.11	0.45	0.42	0.31	0.56	0.26	0.45	0.48	0.38	0.48	0.20	0.46	0.52	1																			
**i17**	0.41	0.39	0.37	0.40	0.51	0.39	0.29	0.50	0.51	0.53	0.49	0.27	0.50	0.52	0.51	0.36	1																		
**i18**	0.34	0.43	0.25	0.55	0.57	0.41	0.34	0.39	0.61	0.62	0.48	0.36	0.31	0.70	0.65	0.49	0.60	1																	
**i19**	0.29	0.39	0.27	0.50	0.56	0.39	0.29	0.42	0.54	0.61	0.48	0.33	0.38	0.60	0.63	0.41	0.50	0.64	1																
**i20**	0.50	0.33	0.29	0.29	0.44	0.47	0.24	0.28	0.34	0.42	0.53	0.24	0.38	0.36	0.39	0.23	0.47	0.39	0.43	1															
**i21**	0.34	0.44	0.23	0.31	0.40	0.23	0.40	0.27	0.32	0.41	0.33	0.41	0.39	0.40	0.39	0.40	0.42	0.42	0.39	0.43	1														
**i22**	0.34	0.42	0.33	0.48	0.55	0.36	0.30	0.49	0.53	0.57	0.46	0.29	0.42	0.63	0.55	0.42	0.65	0.58	0.53	0.41	0.47	1													
**i23**	0.30	0.45	0.22	0.53	0.58	0.45	0.36	0.35	0.55	0.60	0.47	0.36	0.25	0.60	0.67	0.51	0.47	0.62	0.58	0.43	0.45	0.59	1												
**i24**	0.53	0.46	0.38	0.42	0.52	0.34	0.22	0.35	0.40	0.49	0.46	0.20	0.41	0.49	0.44	0.27	0.53	0.50	0.45	0.52	0.38	0.55	0.47	1											
**i25**	0.29	0.48	0.25	0.31	0.41	0.27	0.45	0.27	0.40	0.44	0.34	0.41	0.23	0.37	0.40	0.45	0.35	0.43	0.38	0.31	0.38	0.39	0.41	0.44	1										
**i26**	0.40	0.32	0.48	0.30	0.43	0.27	0.17	0.44	0.40	0.43	0.31	0.19	0.56	0.42	0.42	0.26	0.47	0.40	0.36	0.35	0.39	0.50	0.40	0.47	0.29	1									
**i27**	0.28	0.42	0.27	0.55	0.55	0.42	0.41	0.39	0.60	0.60	0.44	0.32	0.27	0.64	0.62	0.50	0.49	0.64	0.52	0.29	0.35	0.53	0.60	0.41	0.37	0.44	1								
**i28**	0.34	0.39	0.22	0.48	0.53	0.44	0.37	0.36	0.51	0.54	0.39	0.29	0.31	0.53	0.59	0.45	0.44	0.56	0.55	0.42	0.32	0.47	0.57	0.42	0.35	0.39	0.63	1							
**i29**	0.32	0.54	0.24	0.44	0.53	0.39	0.47	0.39	0.48	0.58	0.42	0.45	0.33	0.49	0.54	0.52	0.48	0.53	0.48	0.38	0.48	0.52	0.55	0.43	0.53	0.38	0.55	0.56	1						
**i30**	0.35	0.38	0.25	0.47	0.45	0.51	0.33	0.35	0.44	0.51	0.51	0.28	0.24	0.47	0.49	0.43	0.46	0.50	0.43	0.44	0.32	0.45	0.50	0.45	0.33	0.36	0.56	0.51	0.49	1					
**i31**	0.43	0.45	0.33	0.41	0.54	0.41	0.31	0.40	0.51	0.60	0.44	0.29	0.41	0.55	0.55	0.41	0.56	0.53	0.51	0.45	0.39	0.59	0.53	0.53	0.39	0.47	0.58	0.54	0.52	0.52	1				
**i32**	0.34	0.41	0.28	0.51	0.57	0.39	0.31	0.42	0.58	0.61	0.44	0.33	0.32	0.64	0.61	0.46	0.57	0.64	0.54	0.35	0.35	0.59	0.57	0.46	0.35	0.45	0.66	0.60	0.56	0.52	0.69	1			
**i33**	0.37	0.33	0.29	0.31	0.35	0.58	0.34	0.30	0.36	0.39	0.50	0.27	0.29	0.38	0.40	0.29	0.41	0.33	0.39	0.44	0.25	0.37	0.39	0.45	0.33	0.34	0.40	0.44	0.39	0.49	0.45	0.36	1		
**i34**	0.34	0.53	0.22	0.48	0.54	0.36	0.51	0.30	0.48	0.56	0.40	0.46	0.28	0.51	0.53	0.57	0.46	0.54	0.51	0.33	0.48	0.53	0.54	0.43	0.54	0.38	0.53	0.57	0.74	0.49	0.54	0.57	0.46	1	
**i35**	0.34	0.42	0.28	0.59	0.59	0.45	0.38	0.40	0.55	0.61	0.47	0.33	0.25	0.63	0.61	0.48	0.49	0.63	0.56	0.40	0.37	0.57	0.59	0.45	0.39	0.40	0.70	0.63	0.57	0.58	0.58	0.69	0.47	0.63	1

**Table 2:** Significant differences by school year in the competency domains.

		M (SD)	*F*	*df*	*p*	*n* ^2^	Difference in Means
Initiating relationships	1styear	14.71 (5.55)	1.80	4, 1256	0.13	0.006	
	2ndyear	14.08 (5.43)					
	3rdyear	13.86 (5.56)					
	4thyear	15.29 (5.14)					
Assertiveness/ability to say ‘no’	1styear	17.37 (6.85)	2.63	4, 1245	0.03	0.008	4th year-1st year: 2.36 * 4th year-2nd year: 2.31 *
	2ndyear	17.42 (6.17)					
	3rdyear	18.22 (6.13)					
	4thyear	19.73 (5.17)					
Self-disclosure	1styear	12.01 (6.01)	2.55	4, 1249	0.04	0.008	4th year-1st year No differences post-hoc 4th year-2nd year No differences post-hoc
	2ndyear	12.51 (5.34)					
	3rdyear	12.89 (5.42)					
	4thyear	13.97 (5.06)					
Providing emotional support	1styear	16.99 (5.66)	2.77	4, 1272	0.02	0.009	4thyear-1styear: 2.49 * 4thyear-2ndyear: 2.28 *
	2ndyear	17.21 (5.72)					
	3rdyear	17.68 (5.53)					
	4thyear	19.49 (4.05)					
Resolving interpersonal conflicts	1styear	15.78 (5.92)	1.98	4, 1255	0.09	0.006	
	2ndyear	16.02 (5.46)					
	3rdyear	16.26 (5.64)					
	4thyear	18.04 (4.30)					

* *p* < 0.05.

**Table 3:** Mean Scores and Standard Deviation by gender in the competency domains.

AICQc Scales		M (SD)	Total (SD)
Initiating relationships	Boys	14.41 (5.48)	14.30 (5.51)
	Girls	14.20 (5.51)	
Assertiveness/ability to say ‘no’	Boys	16.62 (6.36)	17.77 (6.38)
	Girls	18.96 (6.11)	
Self-disclosure	Boys	12.93 (5.81)	12.55 (5.61)
	Girls	12.15 (5.39)	
Providing emotional support	Boys	16.68 (5.72)	17.39 (5.60)
	Girls	18.12 (5.35)	
Resolving interpersonal conflicts	Boys	15.53 (5.77)	16.10 (5.64)
	Girls	16.72 (5.44)	

## Appendix A

**Table A1:** Adolescent Interpersonal Competence Questionnaire for Courtship(AICQc).

TeSientesCapazde… (*Do You Feel You Are Able to …*)	1 Me SientoTorpe (*I Feel Awkward with This*)	2 No Se Me da Muy Bien (*I Can’t Do This Very Well*)	3 SeMe da Medio Bien (*I Can Do This OK*)	4 Soy Bastante Bueno/a enEsto (*I’m Fairly Good at This*)	5 Soy Muy Bueno/a enEsto (*I’m Very Good at This*)
1.Pedirle a alguien que te gusta o te resulta atractivo hacer algo juntos como ir a ver un partido, o ir al cine? (*Ask someone you like or feel attracted to to do something together like going see a match, or going to the cinema?*)	1	2	3	4	5
2. Decirle a alguien que te gusta o con quien empiezas a salir que no te gusta la forma en que te ha estado tratando? (*Tell someone you like or who you start dating that you don’t like the way they’ve been treating you?*)	1	2	3	4	5
3. Contarle algo íntimo sobre ti a alguien que te interesa o estás conociendo? (*Say something intimate about yourself to someone you are interested in or are getting to know?*)	1	2	3	4	5
4. Ayudar a alguien que te gusta a comprender sus pensamientos y sentimientos para tomar una decisión importante en su vida, por ejemplo, elegir qué quiere estudiar? (*Help someone you like to understand their thoughts and feelings when making an important life-choice, such as choosing what they want to study?*)	1	2	3	4	5
5. Resolver problemas con alguien que te gusta o con quien empiezas a salir de forma que las cosas vayan mejor, en vez de peor? (*Resolve problems with someone you like or who you start dating to help things get better, instead of worse?*)	1	2	3	4	5
6. Hacer todo lo posible por empezar nuevas amistades? (*Do everything possible to start new friendships?*)	1	2	3	4	5
7. Decir “no” cuando alguien que te gusta te pide que hagas algo que no quieres hacer? (*Say “no” when someone you like asks you to do something you don’t want to do?*)	1	2	3	4	5
8. Dejar que alguien que estás conociendo o con quien empiezas a salir vea tu lado más sensible? (*Let someone you are getting to know or starting to date see your most sensitive side?*)	1	2	3	4	5
9. Hacer que alguien que te gusta o con quien empiezas sienta que entiendes sus problemas? (*Make someone you like or start dating feel that you understand their problems?*)	1	2	3	4	5
10. Gestionar los problemas con alguien que te gusta o con quien empiezas a salir de forma que, a la larga, os haga felices a ambos? (*Manage problems with someone you like or who you start dating in a way that, in the long run, makes both of you happy?*)	1	2	3	4	5
11. Hablar y mantener conversaciones con alguien nuevo a quien te gustaría conocer? (*Talk to and have conversations with someone new you would like to know better?*)	1	2	3	4	5
12. Rechazar una petición que no crees que sea adecuada cuando la hace alguien que te gusta o con quien empiezas a salir? (*Refuse a request you don’t think is appropriate when made by someone you like or who you start dating?*)	1	2	3	4	5
13. Contarle algo de ti que te avergüenza a alguien que te gusta o con quien empiezas a salir? (*Tell someone you like or start dating something about yourself which you are ashamed of?*)	1	2	3	4	5
14. Ayudar a alguien que te gusta o con quien empiezas a salir a comprender mejor sus problemas? (*Help someone you like or start dating to understand their problems better?)*	1	2	3	4	5
15. Resolver los problemas con alguien que te gusta o con quien empiezas a salir de forma que ninguno de los dos salga herido o resentido? (*Resolve problems with someone you like or start dating so that neither of you feels hurt or resentful?*)	1	2	3	4	5
16. Defender tus derechos cuando alguien que te gusta o con quien empiezas a salir es grosero o no los respeta? (*Stand up for your rights when someone you like or start dating is rude or disrespectful to you?*)	1	2	3	4	5
17. Abrirte a alguien que te gusta o con quien empiezas a salir y dejar que te conozca de verdad? (*Open up to someone you like or start dating and let them know the real ‘you’?*)	1	2	3	4	5
18. Ayudar a alguien que te gusta o con quien empiezas a salir a enfrentarse a la presión o a momentos difíciles? (*Help someone you like or start dating to cope with pressure or difficulties?*)	1	2	3	4	5
19. Tratar los problemas con alguien que te gusta o con quien empiezas a salir de forma que no siempre salga perdiendo la misma persona? (*Resolve problems with someone you like or start dating in a way that the same person doesn’t always lose out?*)	1	2	3	4	5
20. Presentarte tú mismo/a a alguien que te gustaría conocer o con quien te gustaría salir? (*Introduce yourself to someone you’d like to meet or who you’d like to date?*)	1	2	3	4	5
21. Decirle a alguien que te gusta o con quien empiezas a salir que está haciendo algo que te avergüenza? (*Tell someone you like or start dating that they’re doing something that embarrasses you?*)	1	2	3	4	5
22. Compartir con alguien que te gusta o con quien empiezas a salir tus ideas y sentimientos? (*Share with someone you like or start dating your ideas and feelings?*)	1	2	3	4	5
23. Resolver los conflictos con alguien que te gusta o con quien empiezas a salir antes de que se conviertan en peleas? (*Resolve conflicts with someone you like or start dating before they turn into arguments?*)	1	2	3	4	5
24. Llamar por teléfono a alguien que te atrae o con quien empiezas a salir para quedar para veros y hacer algo? (*Phone someone you are attracted to or you are starting to date to meet up and do something together?*)	1	2	3	4	5
25. Enfrentarte a alguien que te gusta o con quien empiezas a salir porque ha roto una promesa? (*Challenge someone you like or start dating because they’ve broken a promise?*)	1	2	3	4	5
26. Contarle a alguien que te gusta o con quien empiezas a salir cosas que no quieres que sepa todo el mundo? (*Tell someone you like or start dating things you don’t want everyone to know?*)	1	2	3	4	5
27. Hacer que alguien que te gusta o con quien empiezas a salir se sienta mejor cuando está triste o deprimido? (*Help someone you like or start dating to feel better when they are sad or depressed?*)	1	2	3	4	5
28. Superar rápidamente los problemas con alguien que te gusta o con quien empiezas a salir? (*Quickly get over issues with someone you like or start dating?*)	1	2	3	4	5
29. Decirle a alguien que te gusta o con quien empiezas a salir que ha hecho algo que ha herido tus sentimientos? (*Tell someone you like or are starting to date that they’ve done something which has hurt your feelings?*)	1	2	3	4	5
30. Dar una buena primera impresión cuando conoces a alguien con quien te gustaría comenzar una amistad o empezar a salir? (*Make a good first impression on someone with whom you’d like to start a friendship or start dating?*)	1	2	3	4	5
31. Decirle a alguien que te gusta o quien empiezas a salir cuánto lo aprecias o te importa? (*Tell someone you like or who you start dating how much you appreciate or value them?*)	1	2	3	4	5
32. Mostrar que realmente te interesa o te importa cuando alguien que te gusta o con quien empiezas a salir te cuenta sus problemas? (*Show that you are really interested and really care when someone you like or start dating tells you about their problems?*)	1	2	3	4	5
33. Ir a fiestas o actividades con gente nueva para hacer nuevos amigos o conocer a más gente? (*Go to parties or activities with new people to make new friends or meet more people?*)	1	2	3	4	5
34. Decirle a alguien que te gusta o con quien empiezas a salir que ha hecho algo que te ha hecho enfadar? (*Tell someone you like or are starting to date when they’ve done something that has made you angry?*)	1	2	3	4	5
35. Dar consejos que puedan ayudar a alguien que te gusta o con quien empiezas a salir? (*Give advice that could help someone you like or start dating?*)	1	2	3	4	5
